# Moderate Physical Activity in Healthy Adults Is Associated With Cardiac Remodeling

**DOI:** 10.1161/CIRCIMAGING.116.004712

**Published:** 2016-08-16

**Authors:** Timothy J.W. Dawes, Ben Corden, Sorcha Cotter, Antonio de Marvao, Roddy Walsh, James S. Ware, Stuart A. Cook, Declan P. O’Regan

**Affiliations:** From the MRC Clinical Sciences Centre (CSC), London, United Kingdom (T.J.W.D., B.C., A.d.M., J.S.W., S.A.C., D.P.O.); Division of Experimental Medicine, Department of Medicine, Imperial College London, United Kingdom (S.C.); NIHR Royal Brompton Cardiovascular Biomedical Research Unit and the National Heart & Lung Institute at Imperial College London, United Kingdom (R.W., J.S.W., S.A.C.); National Heart Centre and Duke–National University of Singapore (S.A.C.).

**Keywords:** cardiac magnetic resonance imaging, exercise physiology, left ventricular remodeling, remodeling, right ventricle

## Abstract

**Background—:**

Cardiac mass and volumes are often elevated in athletes, but it is not known whether moderate physical activity is also associated with cardiac dilatation and hypertrophy in a healthy adult population.

**Methods and Results—:**

In total, 1096 adults (54% female, median age 39 years) without cardiovascular disease or cardiomyopathy-associated genetic variants underwent cardiac magnetic resonance imaging to determine biventricular volumes and function. Physical activity was assessed using a validated activity questionnaire. The relationship between cardiac parameters and activity was assessed using multiple linear regression adjusting for age, sex, race, and systolic blood pressure. Logistic regression was performed to determine the effect of activity on the likelihood of subjects having cardiac dilatation or hypertrophy according to standard cardiac magnetic resonance normal ranges. Increasing physical activity was associated with greater left ventricular (LV) mass (β=0.23; *P*<0.0001) and elevated LV and right ventricular volumes (LV: β=0.26, *P*<0.0001; right ventricular: β=0.26, *P*<0.0001). Physical activity had a larger effect on cardiac parameters than systolic blood pressure (0.06≤β≤0.21) and a similar effect to age (−0.20≤β≤−0.31). Increasing physical activity was a risk factor for meeting imaging criteria for LV hypertrophy (adjusted odds ratio 2.1; *P*<0.0001), LV dilatation (adjusted odds ratio 2.2; *P*<0.0001), and right ventricular dilatation (adjusted odds ratio 2.2; *P*<0.0001).

**Conclusions—:**

Exercise-related cardiac remodeling is not confined to athletes, and there is a risk of overdiagnosing cardiac dilatation or hypertrophy in a proportion of active, healthy adults.

## Abstract 2

Supplemental Digital Content is available in the text.

Cardiac chamber enlargement and the capacity to increase cardiac output are hallmarks of athletic training.^1^ Typical adaptations of the heart to sustained exercise are biventricular enlargement, enhanced early diastolic filling, and bi-atrial dilatation.^2^ The majority of athletes develop relatively mild adaptations, and the current view of athlete’s heart is one of adaptive physiology and not preclinical disease.^1^ The benefits of moderate exercise are well recognized,^3^ and public health organizations recommend that adults should engage in 150 minutes of moderate-intensity exercise per week.^4^ In recent years, athletic participation has more than doubled in all major demographic groups.^5^ Although myocardial remodeling has been documented across a spectrum of physical conditioning from recreational marathon runners to elite competitive endurance athletes,^6,7^ the prevalence of cardiac dilatation at mild to moderate levels of physical activity is not known. Misclassification of cardiac disease in healthy adults may lead to erroneous risk stratification, inappropriate management, and unnecessary further investigations. In this study, we examined a healthy adult population without cardiovascular risk factors or genetic variants associated with cardiac disease to determine if typical levels of physical activity were associated with cardiac remodeling.

**See Editorial by Baggish**

**See Clinical Perspective**

## Methods

### Subjects

One thousand one hundred fifty-eight healthy adult volunteers (54% female; median age 39.2 years; range 18–97 years) were prospectively recruited by newspaper advertisement as part of the UK Digital Heart Project (www.digital-heart.org) between March 2011 and January 2015. We excluded participants at screening who had known cardiovascular disease or were being treated for hypertension, diabetes mellitus, or hypercholesterolemia, or had a first degree relative with cardiomyopathy. All subjects were sequenced for disease-causing cardiomyopathy and channelopathy genes using a comprehensive sequencing assay,^8^ and 62 individuals with putative pathogenic genetic variants were excluded.^9^ Female subjects were excluded if they were pregnant or breastfeeding but were eligible if they took oral contraceptives. Standard published safety contraindications to magnetic resonance imaging were applied.^10^ All subjects provided written informed consent for participation in the study, which was approved by a research ethics committee.

### Physical Activity Survey

Physical activity grading was based on the Copenhagen City Heart Study Leisure Time Physical Activity Questionnaire, performed on the same day as the cardiac magnetic resonance imaging. This questionnaire has been shown to discriminate subjects with respect to maximal oxygen uptake and to predict mortality.^3,11^ Categories of activity were based on participants’ typical level of activity over the preceding 12 months and were defined as follows: level I, almost entirely sedentary with no regular exercise; level II, light physical activity or exercise for 1 to 3 hours per week; level III, moderate physical activity or exercise between 3 and 5 hours per week; and level IV, >5 hours exercise per week or regular competitive sports (Table I in the Data Supplement).

### Body Composition and Blood Pressure

All measurements were performed by trained research nurses at the study center. Height and weight were measured without shoes while wearing scrubs. Total body fat mass and lean mass were measured using bioelectrical impedance (InBody 230, BioSpace, Los Angeles, CA).^12^ Blood pressure (BP) was measured after 5 minutes rest in accordance with European Society of Hypertension guidelines^13^ using a calibrated oscillometric device (Omron M7, Omron Corporation, Kyoto, Japan).

### Magnetic Resonance Imaging Protocol

**Figure 1.**
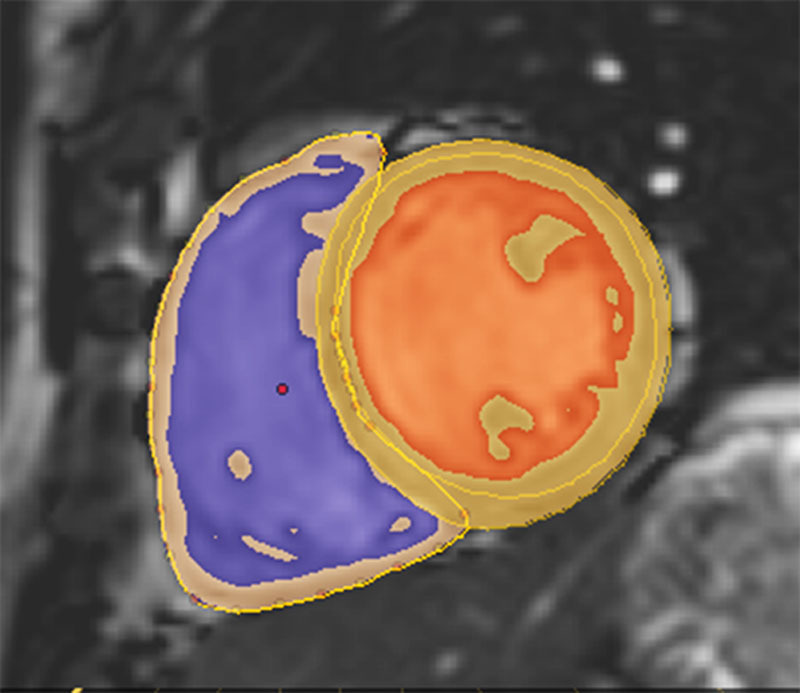
A short-axis cine image demonstrating the assessment of biventricular volumes and function in a healthy adult (right ventricular cavity in purple, left ventricular cavity in orange, and myocardium in yellow with epicardial contours defining the left and right ventricles).

Cardiac magnetic resonance (CMR) imaging was performed at a single site on a 1.5T Philips Achieva (Best, Netherlands). A standard clinical protocol for assessing biventricular function and volumes was followed according to published international guidelines.^14^ Images were stored on an open-source database (MRIdb, Imperial College London, UK).^15^ Volumetric analysis of the cine images was performed using CMRtools (Cardiovascular Imaging Solutions, London, UK) following a standard protocol (Figure 1).^16^ All measurements were obtained from the short-axis stack and used valve tracking on a corresponding long-axis cine. Papillary muscle and trabeculations were included in the mass measurement. Volumes and mass were indexed to body surface area calculated using the Mosteller formula and were classified as dilated or nondilated on the basis of indexed normal ranges stratified by age and sex, as recommended in Society of Cardiac Magnetic Resonance (SCMR) guidelines.^16^ These reference ranges are the 95% confidence intervals in an independent healthy adult population obtained using identical cardiac software and analysis methods.^17,18^ Indexed volumetric data were left ventricular (LV) mass (LVMi), LV and right ventricular (RV) end-diastolic volumes (LVEDVi and RVEDVi), LV and RV end-systolic volumes, LV and RV stroke volumes (LVSVi and RVSVi), and LV and RV ejection fractions. Cardiac index (CI) was derived as (LVSV×heart rate [HR]) divided by body surface area. Interstudy and interobserver reproducibility were assessed in 20 subjects.

### Statistical Analysis

Data were analyzed in R (www.r-project.org) using RStudio Server (Boston, MA). Categorical variables were expressed as percentages. Because of skewed distributions, continuous variables were expressed as median±interquartile range. Inter-rater reliability was assessed using Krippendorff’s alpha coefficient and interstudy agreement using Kendall’s Tau coefficient. Confidence intervals were calculated using 10 000 bootstrap samples.

The Jonckheere–Terpstra test for ordered alternatives was used to assess if there was a statistically significant trend of higher median LVMi, LVEDVi and RVEDVi, indexed LV end-systolic volume and indexed RV end-systolic volume, LV concentricity, LV SVi and RV SVi, CI, and lower median HR and EF, with higher levels of physical activity. The differences between other physical and demographic parameters with respect to activity was assessed with the Kruskal–Wallis H Test. Differences between each individual activity level were then assessed using post hoc pairwise Wilcoxon tests, Bonferroni-corrected for multiple comparisons (a corrected *P* value of <0.05 was taken to be significant).

Multiple linear regression was used to assess the association between activity and continuous cardiac variables. Separate models were developed using each cardiac parameter as the outcome variable (LVMi, HR, CI, LVEDVi and RVEDVi, SVi, and EF). The independent variable was physical activity with covariates of age, sex, ethnicity, and systolic BP. Ethnicity was dummy-coded with the largest group, white, as the reference.

The associations between activity level and categorical variables (presence/absence of dilatation or hypertrophy) were assessed with Chi-squared tests and logistic regression models, again adjusted for age, sex, race, and systolic BP.

Regression models satisfied the assumptions of linear regression. In all tests, a *P* value <0.05 was taken as significant. Standardized β-coefficients are reported in the text, and both standardized and unstandardized β-coefficients are provided in the tables.

## Results

All data sets were analyzed and included in the final analysis. Subject characteristics are described in Table 1. Cardiac volumes and function are summarized in Table 2. Reliability of cardiac volumetry is given in Table II in the Data Supplement.

**Table 1.**
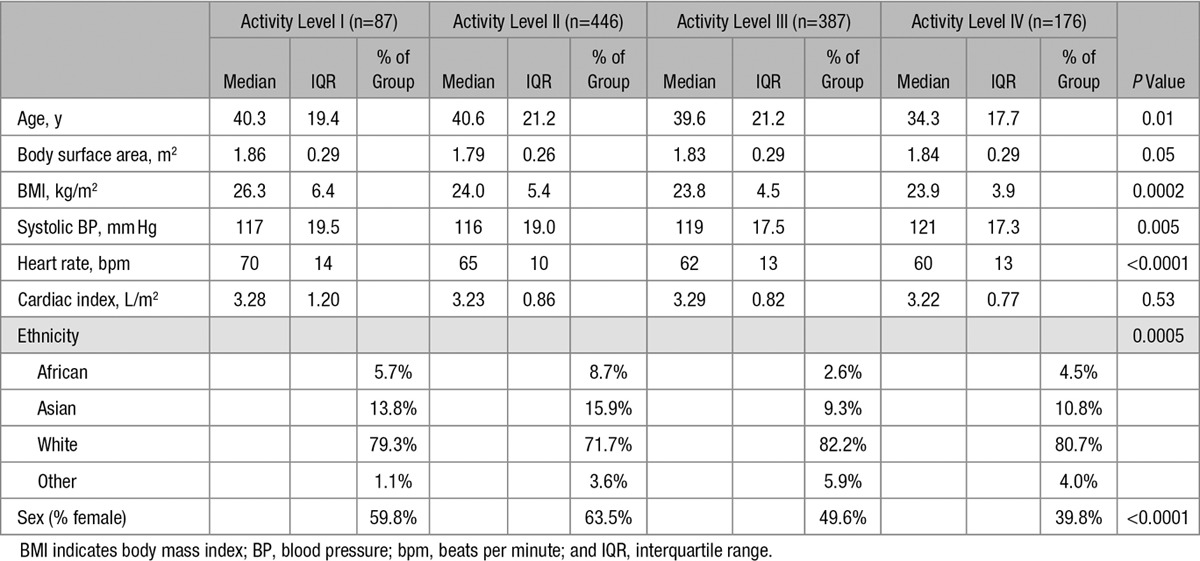
Subject Characteristics (n=1096)

**Table 2.**
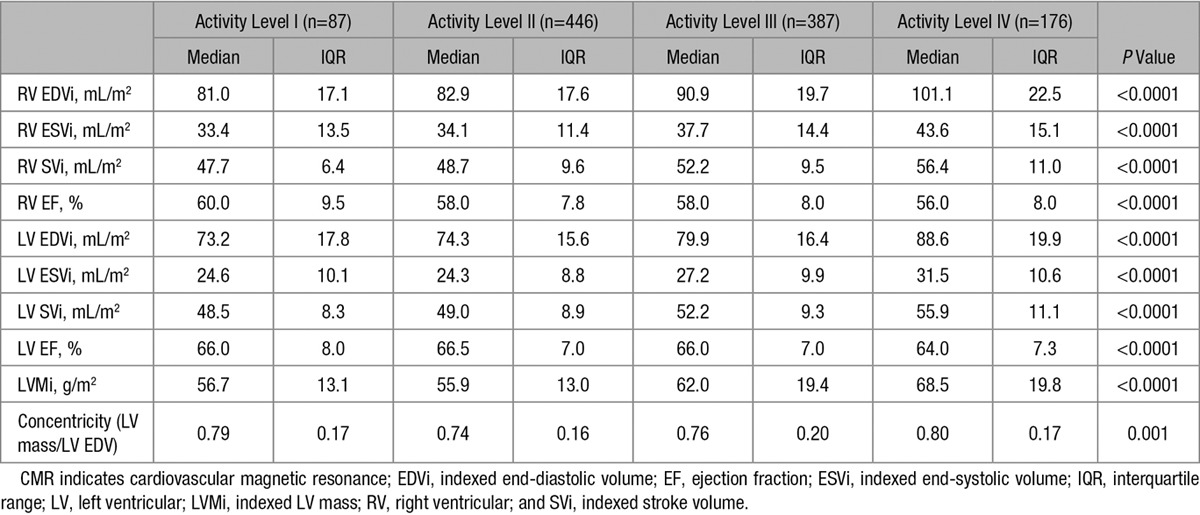
CMR-Derived Volumetry and Functional Assessments (n=1096)

### Physical Activity

In an average week, 8% of the cohort reported engaging in no regular exercise at all (level I), 41% light activity (level II), 35% moderate physical activity (level III), and 16% >5 hours of exercise per week (level IV). Men reported higher activity levels than women (*P*<0.0001), and there was a reduction in exercise level with increasing age (*P*=0.01).

### Unadjusted Associations Between Activity Level and Ventricular Structure and Function

**Figure 2.**
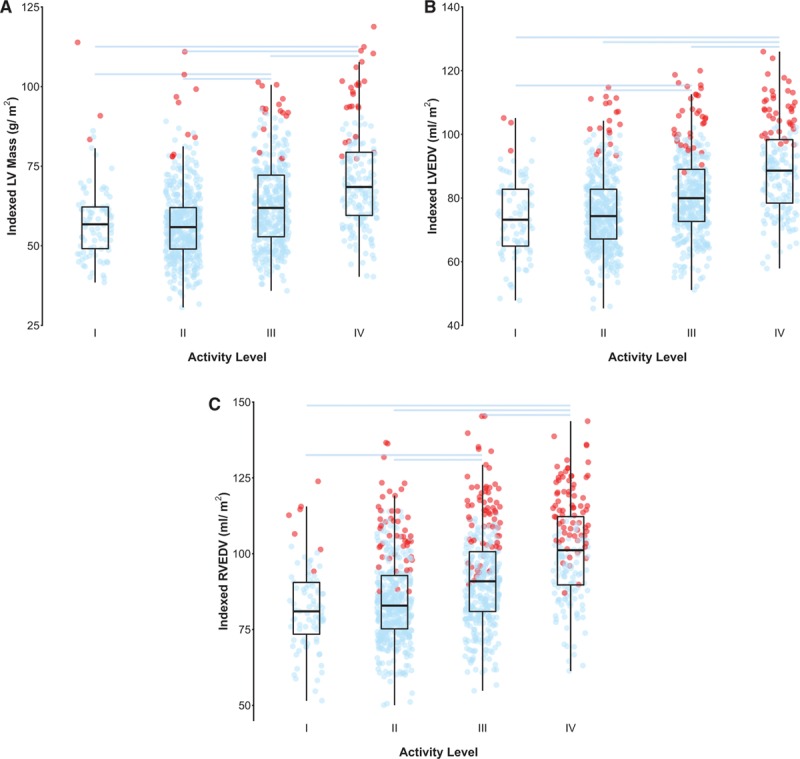
**A**–**C**, Tukey box and whisker plots showing the relationship between activity level on the Copenhagen scale and indexed (**A**) LV mass, (**B**) LVEDV, and (**C**) RVEDV. The jittered dots show data for each subject (n=1096), with blue points indicating a value within the normal range and red points those above the normal range for that individual according to published reference ranges stratified by decade of age and sex.^17,18^ Pair wise comparisons with a Bonferroni-corrected *P* value <0.05 are indicated by horizontal lines. EDV indicates end diastolic volume; LV, left ventricular; and RV, right ventricular.

Table 2 and Figure 2 summarize the associations between activity level and ventricular mass and volumes.

There was a statistically significant pattern of higher LVMi, biventricular volumes, and stroke volumes (LVEDVi, indexed LV end-systolic volume, LVSVi, RVEDVi, indexed RV end-systolic volume, and RVSVi) with higher levels of physical activity (all *P*<0.0001). There was also a significant pattern of increasing LV concentricity (LV mass to LV EDV ratio) with increasing activity (*P*=0.003). Resting HR decreased with increasing activity (*P*<0.0001). Consistent with the increased SV but decreased resting HR, there was no significant association between resting CI and activity level (*P*=0.94). There was a significant pattern of decreasing LV and RV EF as activity level increased (LV EF, *P*<0.0001; RV EF, *P*<0.0001).

### Adjusted Associations Between Activity Level and Ventricular Structure and Function

A summary of the associations between activity level and measures of ventricular structure and function, adjusted for age, sex, ethnicity, and systolic BP, are shown in Table 3. The complete regression models are provided in Table III in the Data Supplement. There was no interaction between sex and activity level for any variable.

**Table 3:**
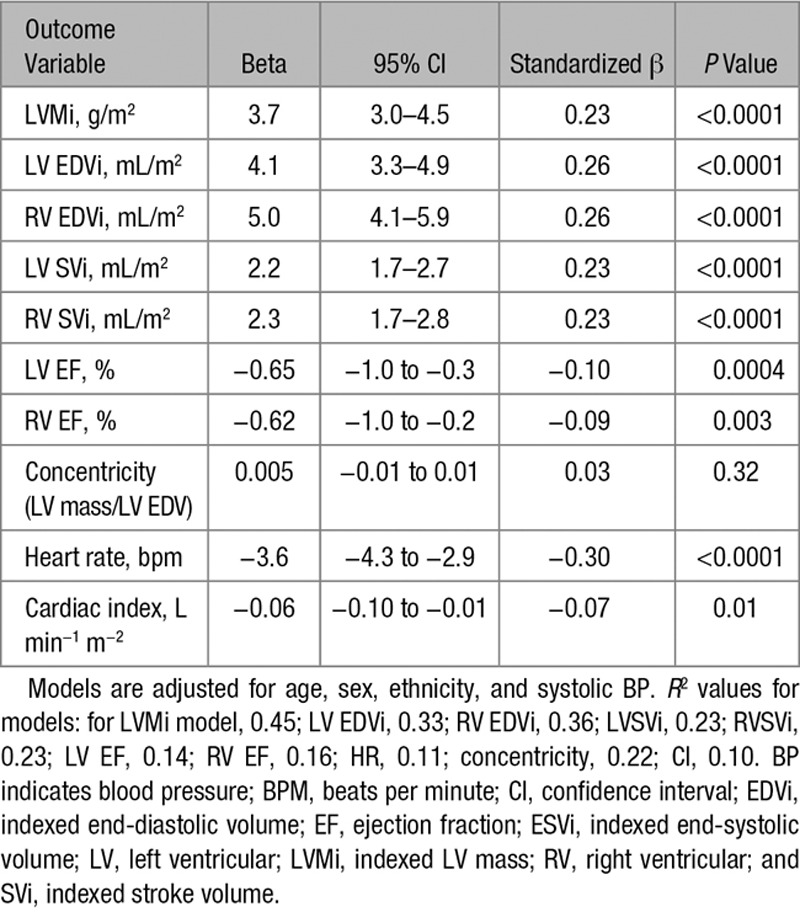
Summary of the Associations Between Activity Level and Measures of Cardiac Structure and Function, Adjusted for Age, Sex, Ethnicity, and Systolic BP

After adjustment for age, sex, ethnicity, and systolic BP, LVMi and biventricular volumes remained positively associated with activity (LVMi, β=0.23; LVEDVi, β=0.26; RVEDVi, β=0.26; all *P*<0.0001). For each increase in activity level (I–IV), LVMi increased by 3.7 g/m^2^, LVEDVi by 4.1 mL/m^2^, and indexed RVEDVi by 5.0 mL/m^2^.

By way of comparison, systolic BP had a weaker association with LVEDVi (β=0.08; *P*=0.005), RVEDVi (β=0.06; *P*=0.03), and LVMi (β=0.21; *P*<0.0001) compared with activity level. Age and activity showed a showed a similar strength of association with cardiac parameters (LVEDVi, β=−0.32, *P*<0.0001; RVEDVi, β=−0.28, *P*<0.0001; LVMi, β=−0.21, *P*<0.0001).

LVSVi and RVSVi were positively associated with activity after adjustment for potential confounders (LVSVi, β=0.23, *P*<0.0001; RVSVi, β=0.23, *P*<0.0001). Each increase in activity level was associated with an increase in LVSVi of 2.2 mL/m^2^ and RVSVi of 2.3 mL/m^2^. HR had a negative association with activity (β=−0.30, *P*<0.0001), and each activity level was associated with a 3.6 bpm decrease in resting HR. Overall, there was a weak negative association of activity and CI (β=−0.07, *P*=0.01). LV and RV ejection fractions showed weak negative associations with increasing activity (LV ejection fraction, β=−0.10, *P*=0.0004; RV ejection fraction, β=−0.09, *P*=0.003). There was no association between concentricity (LV mass to LV EDV ratio) and physical activity after adjustment for potential confounders (β=0.03, *P*=0.34).

### Sensitivity Analyses

To ensure that these results were not driven by a small number of true athletes within the >5 hours per week of exercise (level IV) group, we repeated the multiple regression analyses with the exclusion of the highest activity level group and found a similar pattern of remodeling in the level III subjects compared with the other participants. We also compared ratiometric scaling with allometric adjustment for height^2.7^, as well as adjustment by body composition and height, and confirmed that this did not influence the pattern of findings.^19^ Finally, we repeated the analyses with HR, history of current smoking, and history of previous smoking as additional covariates and found the pattern of the results unchanged (data for all sensitivity analyses are given in Tables IV–VII in the Data Supplement).

### Classification by Normal Ranges

**Figure 3.**
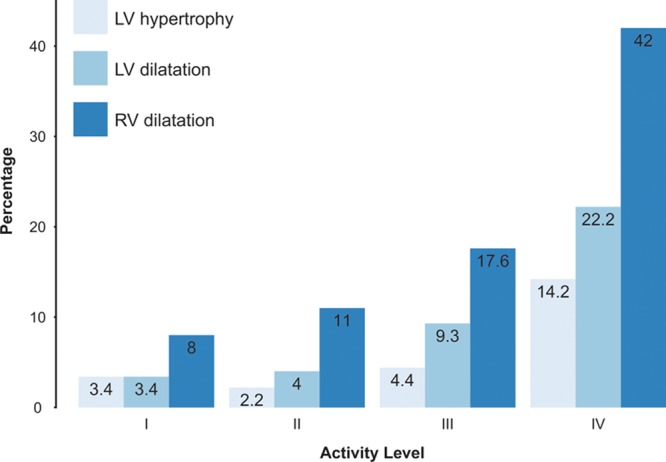
Bar chart showing percentage of subjects classed as having LV hypertrophy, LV dilatation, or RV dilatation grouped by activity level on the Copenhagen scale (ie, a LVMi, LVEDVi, or RVEDVi above the normal range for that individual according to published reference ranges stratified by decade of age and sex^17,18^). For LV hypertrophy, χ^2^(3)=39.1, *P*<0.0001; for LV dilatation, χ^2^(3)=55.2, *P*<0.0001; for RV dilatation, χ^2^(3)=89.4, *P*<0.0001. EDVi indicates indexed end-diastolic volume; LV, left ventricular; LVMi, indexed LV mass; and RV, right ventricular.

The proportions of participants classified as having LV hypertrophy, LV dilatation, or RV dilatation by CMR criteria^20^ increased with activity level (Figure 3; all *P*<0.0001).

In logistic regression models (adjusting for age, sex, ethnicity, and systolic BP), activity level remained a significant predictor of LV hypertrophy (adjusted odds ratio per activity level =2.1, *P*<0.0001), LV dilatation (adjusted odds ratio 2.2; *P*<0.0001), and RV dilatation (adjusted odds ratio 2.2; *P*<0.0001). There was no significant interaction between sex and activity level (all *P*≥0.28). Table 4 summarizes the logistic regression models.

**Table 4.**
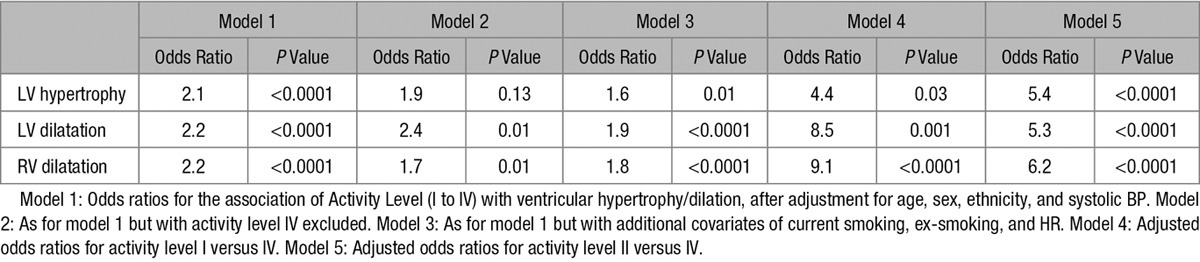
Summary of Logistic Regression Models

As mentioned earlier, to ensure these results were not been driven by a small number of true athletes in the level IV group, we repeated the logistic regression analyses with the exclusion of these subjects. The association of activity with LV and RV dilatation remained significant (adjusted odds ratio 2.4 for LV dilatation [*P*=0.01] and 1.7 for RV dilatation [*P*=0.01]), but the association with LV hypertrophy was no longer significant (adjusted odds ratio 1.9; *P*=0.13).

To further understand the association between activity and ventricular geometry, we compared level I subjects with level IV subjects. The resulting adjusted odds ratio was 4.4 for LV hypertrophy (*P*=0.03), 8.5 for LV dilatation (*P*=0.001), and 9.1 for RV dilatation (*P*<0.0001). Similarly, we compared level II subjects with level IV subjects; the resulting adjusted odds ratio was 5.4 for LV hypertrophy (*P*<0.0001), 5.3 for LV dilatation (*P*<0.0001), and 6.2 for RV dilatation (*P*<0.0001).

Finally, we repeated the logistic regression analyses with HR, history of current smoking, and history of previous smoking as additional covariates and found the pattern of the results unchanged (LV hypertrophy odds ratio 1.6, *P*=0.01; LV dilatation 1.9, *P*<0.0001; RV dilatation 1.8, *P*<0.0001).

## Discussion

In adults with no known comorbidities of cardiovascular disease and no genetic variants associated with cardiomyopathy, increasing physical activity is an independent predictor of elevated biventricular volumes and LV mass. The effect of activity on cardiac structure is greater than that of systolic BP and similar to that of age. Unless the hours of regular physical activity per week are considered during CMR evaluation of the heart, there is a risk of overdiagnosing cardiac dilatation or hypertrophy in a proportion of active healthy adults.

Regular physical exercise is strongly associated with a reduction in all-cause mortality even when comparing moderate activity to a sedentary lifestyle.^21^ Several mechanisms have been proposed for this, including a decrease in myocardial oxygen demand, improved myocardial perfusion, and fewer cardiovascular risk factors, such as hypertension, diabetes mellitus, and obesity.^22^ Physiological adaptations of the heart to exercise are mediated by structural, autonomic, and metabolic mechanisms, which increase cardiac output,^23^ and physical activity is associated with higher end-diastolic volumes and lower resting HR.^24^ In our population, we also found that LV mass and biventricular indexed volumes were positively associated with activity level, although the difference between sedentary and light physical activity groups was not significant. We observed that the RV may be particularly sensitive to training, and previous studies have shown incomplete recovery of the RV after exercise and chronic remodeling resistant to detraining.^25,26^ At least moderate exercise was associated with an increase in SV and a decrease in HR—resulting in no net change to resting CI. A modest decrease in biventricular EF was observed at the highest activity level, a finding shared with competitive athletes.^27^ The relationship between cardiac adaptation and exercise persisted after correction for age, sex, ethnicity, and systolic BP. Lean mass is a strong predictor of LV mass and volume,^19^ and exercise may facilitate body fat loss, as well as increasing fat-free mass.^28^ However, we observed that the relationship between activity and cardiac structure was independent of body composition.

We determined if subjects would be classified with cardiac dilatation or hypertrophy using international guidelines for normal ranges in CMR and ensured equivalence by using comparable sequences and identical image analysis methods. A conservative approach, excluding the most active subjects, demonstrated an odds ratio of 2.4 for LV dilatation and 1.7 for RV dilatation in those engaging in 3 to 5 hours of exercise per week compared with those engaging in <3 hours per week. A more liberal analysis, comparing at least 5 hours exercise per week to sedentary counterparts, gave odds ratios between 4.4 and 9.1 for abnormal cardiac indices. Our findings suggest that the interpretation of volumetric parameters when investigating low-risk adults should consider each patient’s activity level as well as the conventional covariates of age, sex, and body surface area to avoid misdiagnosis of structural heart disease. Biventricular dilatation with an increase in SV are features of the athletic heart, but our findings suggest that this pattern may occur with as little as 3 to 5 hours exercise per week, and >5 hours exercise is associated with a high proportion of adults outside published normal ranges. A normal LV concentricity index is also a feature, which may help to distinguish physiological adaptation to exercise from hypertension or cardiomyopathy.^29^

Our cohort of 1096 adults is ≈10× larger than the reference populations used to derive the recommended normal ranges for the CMR community.^17,18^ The inclusion criteria of asymptomatic adults with no known history of cardiac disease were identical except that we did not confirm a normal serum B-type natriuretic peptide. We recruited healthy volunteers by advertisement, but the published reference groups were drawn exclusively from hospital employees and their relatives in 2006.^20^ This demographic has low to intermediate physical activity and may have under-represented more physically active adults.^30^ Compared with the Multi-Ethnic Study of Atherosclerosis (MESA) surveyed in 2002, our cohort had fewer sedentary adults (8% versus 22%),^24^ and although participation in athletic sports has substantially increased over the previous decade,^5^ the rise in overall population activity has been more modest and shows wide geographical variation.^31^ In contrast to previous studies, participants with genetic variants associated with cardiomyopathy were excluded from this study, ruling out potential genetic confounder effects.^9^

Our study has some limitations. The Copenhagen score is a validated and pragmatic approach that clinicians can readily use for assessing activity level. However, more comprehensive surveys, such as those based on the Cross-Cultural Activity Participation Study, provide a detailed breakdown of physical activities, allowing estimation of the metabolic equivalent level.^32^ Because this was an observational study, we could only assess the association between variables, but interventional studies have confirmed a causal relationship between both exercise and detraining on cardiac structure and function.^33^ We performed the most comprehensive sequencing currently available^8^ and would expect that, if present at all, rare variants outside the genes we sequenced would affect <1 in 25 000 healthy controls.^34^ We also did not use contrast medium or T1 mapping in this study and so do not know if unsuspected fibrosis was present in those with dilated hearts, although without genetic substrate this would not be expected.^35^ We did not include RV mass in our analysis because freewall thickness is challenging to measure accurately in healthy volunteers, and it is not a diagnostic criterion for cardiomyopathy^36^; however, endurance athletes demonstrate a balanced increase in both LV and RV mass, and a similar relationship may be expected in moderate exercise.^2^ Although we do not have outcome data, the beneficial effects of exercise are clear,^37^ and regular physical activity may attenuate adverse age-related changes in cardiac structure and function.^38^ There are reports of extreme exercise being associated with arrhythmias, but it is unlikely that moderate exercise contributes to an arrhythmic substrate in healthy volunteers screened for genetic variants.^39^

In conclusion, activity-related cardiac remodeling is not confined to athletes and develops at moderate levels of physical exercise typical of a healthy adult population. This physiological adaptation should be considered when assessing adults for heart disease by CMR because the effects of exercise on the heart are of equal or greater importance than those of age or BP.

## Acknowledgments

We thank our radiographers Giuliana Durighel, Ben Statton, Marina Quinlan, Catherine Holden, and Alaine Berry, and research nurses Tamara Diamond and Laura Monje Garcia.

## Sources of Funding

The study was supported by a Wellcome Trust-GSK Fellowship Grant, by the Medical Research Council, UK, grants from the Fondation Leducq, and by a British Heart Foundation, UK, project grant (PG/12/27/29489). The research was also cofunded by the National Institute for Health Research (NIHR) Biomedical Research Centre based at Imperial College Healthcare NHS Trust, Biomedical Research Unit in Cardiovascular Disease at Royal Brompton & Harefield NHS Foundation Trust and Imperial College London. The views expressed are those of the author(s) and not necessarily those of the NHS, the NIHR, or the Department of Health.

## Disclosures

None.

## Supplementary Material

## CLINICAL PERSPECTIVE

Cardiac chamber enlargement is recognized as a benign, physiological adaptation to sustained athletic training. A common dilemma for clinicians is how to interpret the presence of relatively mild, biventricular dilation or hypertrophy in patients who are not competitive or endurance athletes but do engage in regular moderate physical activity. We studied the relationship between activity and cardiac remodeling in a cohort of over 1000 adults who had undergone screening for known cardiovascular disease and comprehensive sequencing for disease-causing cardiomyopathy genes. We found that increasing activity predicted cardiac volumes and left ventricular mass independently of age, sex, systolic blood pressure, and body surface area. Using published normal ranges, adults engaging in 5 or more hours exercise a week had an odds ratio of between 4.4 and 9.1 for abnormal cardiac indices—with right ventricular dilatation seen in 44%, left ventricular dilatation in 22%, and left ventricular hypertrophy in 14%. Even 3 to 5 hours exercise a week was associated with mild cardiac enlargement—a relationship that was unaffected by heart rate or smoking status. Standard normal ranges have been largely derived from sedentary populations and do not account for the effect of activity, which is at least as influential on cardiac volumes as age. We found that a validated 4-point scale can objectively quantify a patient’s physical activity to guide clinical interpretation of volumetric data. Our study suggests that cardiac chamber enlargement, without a change in left ventricular concentricity index, occurs in a proportion of healthy adults engaging in moderate exercise.
