# Supplementary material for: Moderate Physical Activity in Healthy Adults Is Associated With Cardiac Remodeling
Source: Circ Cardiovasc Imaging. 2016 Aug 16;9(8):e004712. doi: 10.1161/CIRCIMAGING.116.004712 (PMC4991346; doi:10.1161/CIRCIMAGING.116.004712)
Supplement: Supplementary file 1 [file hci-9-e004712-s001.pdf]

# Supplementary material

**Supplementary Table 1:** Participant questionnaire

| | |
|---|---|
| **Do you smoke?** | Yes ☐ No ☐ Ex smoker ☐ |
| **If yes, how long have you smoked for and how many per day?** | _____ years ________ cigarettes per day |
| **If you are an ex-smoker, for how many years did you smoke for? (approximate)** | Number of years: _________ cigarettes per day ____<br>Start date: __/__/____ End date: __/__/____ |
| **Current alcohol consumption:**<br>(1 unit is equal to half a pint of beer, a small glass of wine, or shot of spirit) | ______ units per week |
| **List any medication you now take:** | |
| **Have you had any heart related health problems or symptoms:** | |
| **Please list your past medical and surgical history:** | |
| **Family History of disease:** | |

| | | | |
|---|---|---|---|
| **Height (cm)** | | **Weight** (Kg): | |
| **Pulse rate:** | baseline:<br><br>during scan: | **BP** | 1: ______________<br>2: ______________<br>3: ______________ |
| **Body Fat Mass:** | **Kg** | **Fat Free Mass:** | **Kg** |
| **Medication:** | | | |
| **Activity Score:** | **Physical activity in leisure time over the last 12 months:**<br>1 Almost entirely sedentary with no regular exercise (eg reading or watching television)<br>2 Light physical activity or exercise for 1 to 3 hours per week<br>3 Moderate physical activity or exercise between 3 to 5 hours per week (eg brisk walking, fast cycling, sports that cause perspiration).<br>4 More than 5 hours vigorous exercise per week or regular competitive sports each week | | |

**Supplementary table 2:** Reliability of CMR measurements. Inter-observer agreement is given by Krippendorff's alpha, and inter-study agreement by Kendall’s Tau with 95% confidence intervals.

| | **Inter-observer** | **Inter-study** |
|---|---|---|
| LV EDVi (ml/$m^2$) | 0.99 (0.98 – 0.99) | 0.92 (0.72 – 0.99) |
| LV ESVi (ml/$m^2$) | 0.95 (0.91 – 0.99) | 0.86 (0.65 – 0.94) |
| LV SVi (ml/$m^2$) | 0.98 (0.96 – 0.99) | 0.90 (0.72 – 0.98) |
| LV EF (%) | 0.77 (0.47 – 0.95) | 0.74 (0.45 – 0.86) |
| LVMi (g/$m^2$) | 0.98 (0.96 – 0.99) | 0.96 (0.86 – 0.99) |
| RV EDVi (ml/$m^2$) | 0.98 (0.96 – 0.99) | 0.89 (0.74 – 0.98) |
| RV ESVi (ml/$m^2$) | 0.90 (0.84 – 0.96) | 0.82 (0.60 – 0.92) |
| RV SVi (ml/$m^2$) | 0.97 (0.96 – 0.99) | 0.86 (0.70 – 0.95) |
| RV EF (%) | 0.67 (0.39 – 0.88) | 0.74 (0.43 – 0.89) |

CMR = Cardiovascular Magnetic Resonance; LV = Left ventricular; RV = Right ventricular; EDVi = Indexed end diastolic volume; ESVi = Indexed end systolic volume; SVi = Indexed stroke volume; EF = Ejection fraction; LVMi = Indexed LV mass.

**Supplementary table 3.** Complete multiple linear regression models showing the associations of age, gender, race, systolic blood pressure and activity level with each cardiac MR parameter.

| **Outcome Variable**<br>Independent variables | **Beta** | **Standardised β** | ***p*** |
|---|---|---|---|
| Model 1: LV mass / BSA | | | |
| Age | -0.21 | -0.20 | <.0001 |
| Gender | 12.9 | 0.46 | <.0001 |
| Race: C v AF | 3.3 | 0.05 | .02 |
| Race: C v Asian | -6.7 | -0.16 | <.0001 |
| Race: C v Other | -5.5 | -0.08 | <.0001 |
| Systolic BP | 0.21 | 0.21 | <.0001 |
| Activity level | 3.7 | 0.23 | <.0001 |
| Model 2: LV EDV / BSA | | | |
| Age | -0.32 | -0.32 | <.0001 |
| Gender | 7.6 | 0.28 | <.0001 |
| Race: C v AF | -4.4 | -0.08 | .003 |
| Race: C v Asian | -8.7 | -0.22 | <.0001 |
| Race: C v Other | -5.0 | -0.08 | .002 |
| Systolic BP | 0.07 | 0.08 | .005 |
| Activity level | 4.1 | 0.26 | <.0001 |
| Model 3: RV EDV / BSA | | | |
| Age | -0.34 | -0.28 | <.0001 |
| Gender | 12.1 | 0.37 | <.0001 |
| Race: C v AF | -2.8 | -0.04 | 0.1 |
| Race: C v Asian | -8.5 | -0.18 | <.0001 |
| Race: C v Other | -4.3 | -0.05 | .03 |
| Systolic BP | 0.07 | 0.06 | .03 |
| Activity level | 5.0 | 0.26 | <.0001 |
| Model 4: LV Stroke Volume / BSA | | | |
| Age | -0.14 | -0.24 | <.0001 |
| Gender | 2.7 | 0.17 | <.0001 |
| Race: C v AF | -3.2 | -0.09 | .0005 |
| Race: C v Asian | -5.1 | -0.22 | <.0001 |
| Race: C v Other | -2.7 | -0.07 | .01 |
| Systolic BP | 0.08 | 0.14 | <.0001 |
| Activity level | 2.16 | 0.23 | <.0001 |
| Model 5: RV Stroke Volume / BSA | | | |
| Age | -0.13 | -0.22 | <.0001 |
| Gender | 3.4 | 0.20 | <.0001 |
| Race: C v AF | -3.6 | -0.10 | .0004 |
| Race: C v Asian | -5.5 | -0.21 | <.0001 |
| Race: C v Other | -2.3 | -0.05 | .046 |
| Systolic BP | 0.08 | 0.14 | <.0001 |
| Activity level | 2.3 | 0.23 | <.0001 |
| Model 6: Heart Rate | | | |
| Age | -0.07 | -0.08 | .006 |
| Gender | -2.5 | -0.12 | .0001 |

| | | | |
|---|---|---|---|
| Race: C v AF | -2.6 | -0.06 | .048 |
| Race: C v Asian | 0.17 | 0.006 | .85 |
| Race: C v Other | 1.02 | 0.02 | .48 |
| Systolic BP | 0.08 | 0.12 | .0004 |
| Activity level | -3.6 | -0.30 | <.0001 |
| Model 7: Cardiac Index | | | |
| Age | -0.01 | -0.25 | <.0001 |
| Gender | 0.04 | 0.03 | .32 |
| Race: C v AF | -0.33 | -0.12 | <.0001 |
| Race: C v Asian | -0.32 | -0.16 | <.0001 |
| Race: C v Other | -0.11 | -0.03 | .26 |
| Systolic BP | 0.01 | 0.20 | <.0001 |
| Activity level | -0.06 | -0.07 | .01 |
| Model 8: LV Ejection Fraction | | | |
| Age | 0.08 | 0.20 | <.0001 |
| Gender | -2.8 | -0.26 | <.0001 |
| Race: C v AF | -0.43 | -0.02 | 0.51 |
| Race: C v Asian | 0.66 | 0.04 | 0.16 |
| Race: C v Other | 0.89 | 0.03 | .24 |
| Systolic BP | 0.04 | 0.12 | .0002 |
| Activity level | -0.65 | 0.10 | .0004 |
| Model 9: RV Ejection Fraction | | | |
| Age | 0.06 | 0.14 | <.0001 |
| Gender | -4.0 | -0.33 | <.0001 |
| Race: C v AF | -2.5 | -0.10 | .0007 |
| Race: C v Asian | -0.78 | -0.04 | .14 |
| Race: C v Other | 0.04 | 0.001 | .97 |
| Systolic BP | 0.07 | 0.16 | <.0001 |
| Activity level | -0.62 | -0.09 | .003 |

BSA = Body surface area; LV = Left ventricular; RV = Right ventricular; EDV = End diastolic volume; ESV = End systolic volume; BP = Blood pressure; C = Caucasian; AF = African.

**Supplementary table 4.** Summary of multiple linear regression models with activity level as the predictor, excluding the highest activity level (i.e. comparing <3hrs exercise per week' to '3 to 5hrs exercise per week'. Models adjusted for age, gender, ethnicity and systolic BP.

| **Outcome Variable** | **Beta** | **Standardised β** | ***p*** |
|---|---|---|---|
| LVMi (g/m$^2$) | 4.04 | 0.16 | <.0001 |
| LV EDVi (ml/m$^2$) | 3.78 | 0.15 | <.0001 |
| RV EDVi (ml/m$^2$) | 4.54 | 0.15 | <.0001 |
| LV SVi (ml/m$^2$) | 2.38 | 0.16 | <.0001 |
| RV SVi (ml/m$^2$) | 2.67 | 0.16 | <.0001 |
| LV EF (%) | -0.23 | -0.02 | .44 |
| RV EF (%) | -0.15 | -0.01 | .69 |
| Heart Rate (bpm) | -3.02 | -0.15 | <.0001 |
| Cardiac Index (L min$^{-1}$ m$^{-2}$) | 0.003 | 0.002 | .94 |

LV = Left ventricular; RV = Right ventricular; EDVi = Indexed end diastolic volume; ESVi = Indexed end systolic volume; SVi = Indexed stroke volume; EF = Ejection fraction; LVMi = Indexed LV mass.

**Supplementary table 5.** Summary of multiple linear regression models with raw outcome variables (i.e. not indexed to BSA) but with body composition variables (lean mass, fat mass and height) added to the model. Raw and standardised beta values are presented for the predictor ‘Activity level’, adjusted for age, gender, ethnicity, systolic BP, lean mass, fat mass and height.

| **Outcome Variable** | **Beta** | **Standardised β** | ***p*** |
|---|---|---|---|
| LVM (g) | 5.3 | 0.14 | <.0001 |
| LV EDV (ml) | 5.6 | 0.15 | <.0001 |
| RV EDV (ml) | 6.8 | 0.15 | <.0001 |
| LV SV (ml) | 3.0 | 0.13 | <.0001 |
| RV SV (ml) | 3.2 | 0.13 | <.0001 |
| Cardiac Output (L) | -0.16 | -0.09 | .0005 |

LV = Left ventricular; RV = Right ventricular; EDV = End diastolic volume; ESV = End systolic volume; SV = Stroke volume; EF = Ejection fraction; LVM = LV mass.

**Supplementary table 6.** Summary of multiple linear regression models with outcome variables indexed to height$^{2.7}$, rather than BSA. Lean mass and fat mass are included in the predictors. Raw and standardised beta values are given for the predictor 'Activity level', adjusted for age, gender, ethnicity, systolic BP, lean mass and fat mass.

| **Outcome Variable** | **Beta** | **Standardised β** | ***p*** |
|---|---|---|---|
| LVMi (g/m$^{2.7}$) | 1.4 | 0.19 | <.0001 |
| LV EDVi (ml/m$^{2.7}$) | 1.5 | 0.23 | <.0001 |
| RV EDVi (ml/m$^{2.7}$) | 1.8 | 0.23 | <.0001 |
| Septal Thickness / BSA (mm/m$^{2.7}$) | 0.02 | 0.04 | .18 |
| LV SVi (ml/m$^{2.7}$) | 0.8 | 0.19 | <.0001 |
| RV SVi (ml/m$^{2.7}$) | 0.8 | 0.19 | <.0001 |
| Cardiac Index (L min$^{-1}$ m$^{-2.7}$) | -0.03 | -0.09 | .004 |

LV = Left ventricular; RV = Right ventricular; EDVi = Indexed end diastolic volume; ESVi = Indexed end systolic volume; SVi = Indexed stroke volume; EF = Ejection fraction; LVMi = Indexed LV mass.

**Supplementary table 7.** Summary of multiple linear regression models including smoking status and heart rate as additional co-variates. Cardiac volumes and mass are indexed to BSA. Raw and standardised beta values are given for the predictor 'Activity level', adjusted for age, gender, ethnicity, systolic blood pressure, current smoking, history of previous smoking and heart rate.

| **Outcome Variable** | **Beta** | **Standardised β** | ***p*** |
|---|---|---|---|
| LVMi (g/m$^2$) | 2.7 | 0.17 | <.0001 |
| LV EDVi (ml/m$^2$) | 3.1 | 0.20 | <.0001 |
| RV EDVi (ml/m$^2$) | 3.5 | 0.19 | <.0001 |
| LV SVi (ml/m$^2$) | 1.7 | 0.18 | <.0001 |
| RV SVi (ml/m$^2$) | 1.8 | 0.18 | <.0001 |
| LV EF (%) | -0.41 | -0.07 | .03 |
| RV EF (%) | -0.20 | -0.03 | .33 |
| Concentricity (LV mass/LV EDV) | 0.003 | 0.02 | .58 |

LV = Left ventricular; RV = Right ventricular; EDVi = Indexed end diastolic volume; ESVi = Indexed end systolic volume; SVi = Indexed stroke volume; EF = Ejection fraction; LVMi = Indexed LV mass.
